# Responses of Crop Water Use Efficiency to Climate Change and Agronomic Measures in the Semiarid Area of Northern China

**DOI:** 10.1371/journal.pone.0137409

**Published:** 2015-09-03

**Authors:** Jingting Zhang, Wei Ren, Pingli An, Zhihua Pan, Liwei Wang, Zhiqiang Dong, Di He, Jia Yang, Shufen Pan, Hanqin Tian

**Affiliations:** 1 College of Resources and Environmental Science, China Agricultural University, Beijing, 100193, China; 2 Key Ecology and Environment Experimental Station of Ministry of Agriculture for Field Scientific Observation in Hohhot, Wuchuan, Hohhot, 011705, China; 3 Department of Plant & Soil Sciences, College of Agriculture, Food and Environment, University of Kentucky, Lexington, KY, 40506, United States of America; 4 International Center for Climate and Global Change Research, Auburn University, Auburn, AL, 36849, United States of America; Banaras Hindu University, INDIA

## Abstract

It has long been concerned how crop water use efficiency (WUE) responds to climate change. Most of existing researches have emphasized the impact of single climate factor but have paid less attention to the effect of developed agronomic measures on crop WUE. Based on the long-term field observations/experiments data, we investigated the changing responses of crop WUE to climate variables (temperature and precipitation) and agronomic practices (fertilization and cropping patterns) in the semi-arid area of northern China (SAC) during two periods, 1983–1999 and 2000–2010 (drier and warmer). Our results suggest that crop WUE was an intrinsical system sensitive to climate change and agronomic measures. Crops tend to reach the maximum WUE (WUEmax) in warm-dry environment while reach the stable minimum WUE (WUEmin) in warm-wet environment, with a difference between WUEmax and WUEmin ranging from 29.0%-55.5%. Changes in temperature and precipitation in the past three decades jointly enhanced crop WUE by 8.1%-30.6%. Elevated fertilizer and rotation cropping would increase crop WUE by 5.6–11.0% and 19.5–92.9%, respectively. These results indicate crop has the resilience by adjusting WUE, which is not only able to respond to subsequent periods of favorable water balance but also to tolerate the drought stress, and reasonable agronomic practices could enhance this resilience. However, this capacity would break down under impact of climate changes and unconscionable agronomic practices (e.g. excessive N/P/K fertilizer or traditional continuous cropping). Based on the findings in this study, a conceptual crop WUE model is constructed to indicate the threshold of crop resilience, which could help the farmer develop appropriate strategies in adapting the adverse impacts of climate warming.

## Introduction

In China, annual mean surface air temperature has increased by approximately 1.1°C over the last 50 years, with a continuing drying trend in recent 10 years [1, 2]. Meanwhile, the agronomic practices (cultivars, fertilizers, cropping patterns, etc.) continued being developed rapidly over recent 30 years in China [3, 4]. Water use efficiency (WUE) is quantified by the ratio of crop production to evapotranspiration (Eta) [5], which can provide further insight into the ecological functioning of the land surface and ecosystem resilience i.e. the capacity to absorb disturbances and retain the same function, feedbacks, and sensitivity [6] during changing environment conditions. The improvement in our understanding of how climatic and agronomic factors influence crop WUE is essential to develop sustainable management strategies for future climate change mitigation and adaptation.

Substantial studies have been conducted to address the impact of climate change on WUE of plants across global water deficit area. Evidence showed that plant WUE is negatively correlated with annual precipitation; the conservative WUE of plants adapt to the threat of drought by improving WUE in arid and semi-arid areas [7, 8, 9]; higher temperature within a certain range increase WUE [9, 10], but extreme high temperature reducing crop WUE by enhancing crop transpiration and soil evaporation [11, 12]. Yet, most studies were mainly focused on the impact of single climatic factor (e.g. temperature or precipitation) on WUE. Moreover, the effect of climate change on WUE in agriculture system may be overstated without considering the impact of agronomic managements. Current agricultural activities, such as crop species selection, farming measures, fertilizer application, and irrigation, may affect the biophysical and biochemical processes within ecosystems [13, 14, 15, 16] and would potentially alter the patterns of WUE [17, 18]. Analysis of the WUE variation under various field management conditions can further improve our understanding of the underlying mechanisms of crop WUE response to climate change and help predict consequences of climate change on regional crop WUE.

The semi-arid area of northern China (SAC) accounts for about 11% of the arable land in China [12, 19, 20, 21]. This region is an important source of agricultural yields supporting the increasing food demands and plays an important role in ecological system of China due to its vast area, ecological vulnerability and sensitivity to climate change, and its land-surface feedbacks to climate [19, 20, 21]. In the SAC, a significant warming with a continuous drying trend also has been observed from 1980 to 2013 [2, 22, 23, 24, 25]. This warming-drying trend leads to a fall in agriculture production and induces a lot of severe ecological problems such as water and soil loss, desertification, grassland degradation, soil salinity, and land subsidence [26]. The main crops in the SAC are spring wheat (*Triticum aestivum*), naked oat (*Avena chinensis*), potato (*Solanum tuberosum*), maize (*Zea mays*), millet (*Setaria italica(L)Beauv*.), and rapeseed (*Brassica rapa (campestris) L*.). Continuous cropping was replaced by rotation cropping in the SAC, and chemical fertilization increased significantly during 1980–2010, while the cultivars and cultivation methods did not show big difference [3, 4, 27].

In this study, we investigated the impacts of climate change (temperature, precipitation) and agronomic measures (e.g. fertilization, cropping patterns) on crop WUE in two periods, in the late twentieth century (1983–1999) (stage I), and drier, warmer environment in the early twenty-first century (2000–2010) (stage II). It aims to use long-term meteorological observation (1980–2010), fertilizer allocation household survey (50 households), long-term soil moisture monitoring-field (1983–2010) and cropping pattern experiment (2008–2010) to reveal the coupling effect of multiple climatic factors on WUE and seeks to fill the knowledge gap of how agricultural practices affect crop WUE in the SAC. In brief, the objectives of the study are: 1) to characterize the historical changes in climate factors and fertilizer use; 2) to investigate the historical change of crop WUE; and 3) to examine the relative contribution of the warming-drying trend, fertilization, and cropping pattern on crop WUE, respectively.

## Materials and Methods

### Study area and site description

**Fig 1:**
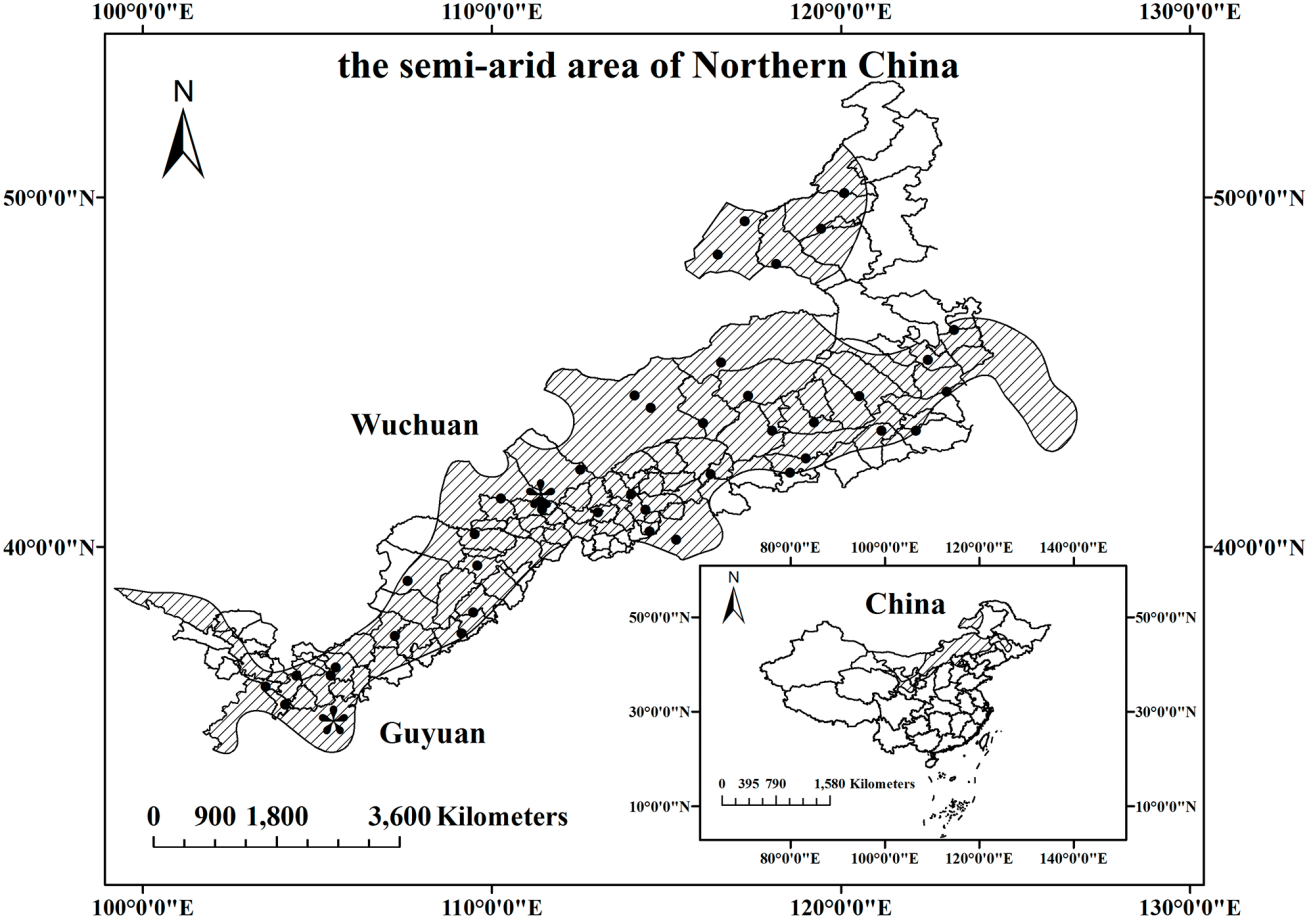
The study region. The semiarid area of northern China (the filled area) and the distribution of the thirty-eight meteorological stations including Wuchuan and Guyuan in the semiarid area of northern China.

The semiarid area of northern China (SAC) (36°01′~49°36′N, 105°45′~124°42′E) (Fig 1), dominated by rain-fed agriculture ecosystems, covers part of Shanxi, Shaanxi, Heilongjiang, Jilin, Inner Mongolia, Ningxia Province and Gansu Province. The SAC is an important ecological transition zone between the farming area (southeastern) and grasslands (northwestern), and also is the most sensitive area to climate change in China [12]. The average temperature in the SAC is about 5.0°C, and the annual precipitation is about 250–500 mm with a large amount of variability. 61.8–72.5% of the annual precipitation happens in July, August and September.

Wuchuan (40°47′~41°23′N, 110°31′~111°52′E) and Guyuan (35°08′~36°22′N, 105°12′~106°34′E) are two the typical regions in SAC [12, 24]. In Wuchuan, the average annual precipitation is about 342 mm with 90% occurs in the crop growing season (May to September), the annual accumulated temperature above 0°C is about 2578°C day. The frost-free period is about 124 d. The soil type is mainly chestnut with high sediment concentrations in the arable layer (Haplic Krastazem, FAO), and the pH value was 8.24–8.27. The contents of total nitrogen, available phosphorus, available potassium, and organic matter in the 0–100 cm soil layer were 1.09 g/kg, 4.96 mg/kg, 105.27 mg/kg, and 1.34%, respectively. The study crops are spring wheat, naked oat, potato, millet, and rapeseed. In Guyuan, the annual precipitation was about 367 mm, of which 65% occurred in crop growing season. The annual mean temperature is 7.9°C. The annual accumulated temperature above 0°C is 2700°C day, and the frost-free period is about 152 d. This area is mainly covered by deep layers of loessal soil and helu soil (Haplic Krastazem, FAO), and the pH value was 8.3–8.5. The contents of total nitrogen, available phosphorus, available potassium, and organic matter in the 0–100 cm soil layer were 1.20 g/kg, 5.82 mg/kg, 151.98 mg/kg, and 2.15%, respectively. The study crops in Guyuan are spring wheat, potato, and maize.

### Data

Historical daily meteorological data (1980–2010) of 38 meteorological stations (Fig 1) was obtained from Chinese Meteorological Administration, including daily and annual mean maximum temperature (Tmax), minimum temperature (Tmin), average temperature (Tave), daily, and annual mean precipitation (P), daily relative humidity (RH), wind speed, sunshine duration, and atmospheric pressure. Firstly, we did the spatial interpolation (Inverse distance) of the meteorological data of 38 stations and then calculated the average climate.

Fertilizer usage of spring wheat, naked oat, potato, and maize from 1983 to 2010 were collected from Wuchuan Agricultural Bureau and Guyuan Agricultural Bureau. The subset of a household survey containing 50 households in Wuchuan and Guyuan is used to calculate the fertilizer applied to different crop types. The amount of fertilizer refers to fertilizer scalar (N+P_2_O_5_+K_2_O).

A long-term monitoring-field data set of the soil moisture content in spring wheat, naked oat, potato, and maize field from 1983 to 2010 was from the Wuchuan Agricultural Meteorology Observation Station and Guyuan Agricultural Meteorology Observation Station. Soil moisture content in different field was measured by gravimetric method at ten soil depths (0–10, 10–20, 20–30, 30–40, 40–50, 50–60, 60–70, 70–80, 80–90, and 90–100 cm) in planting and post-harvest period,was determined. The data set also includes the yields of spring wheat, naked oat, potato, and maize from 1983 to 2010. The experiment was implemented at the fixed crop plot which cultivated the same crop in sequential growing seasons. The management measures (e.g. fertilization) in the monitoring-field experiment were the same to the local management measures.

Crop WUE data set in cropping patterns experiment of millet, rapeseed, and potato was from Wuchuan Scientific Observing and Experimental Station of China Agricultural University (41.60°N, 111.27°E) from 2008 to 2010. Experiment was designed as a two factorial split-plot design with cropping patterns (crop rotation, continuous cropping) as split factors. Crop rotations in this study include millet-potato and rape-potato rotation. Continuous cropping, a cropping system with only one crop growing on the field during one growing season with or without a fallow period, includes millet, rape, and potato in this study. Fertilizer in crop rotation and continuous cropping treatments was applied before planting in each block, including urea (150 kg ha^-1^), diammonium phosphate (90 kg ha^-1^), and potassium chloride (60 kg ha^-1^).Row spacing was 50 cm and each plot area was 6 ×10 m^2^ with a planting density of 6m^-2^ ha^-1^.

### Data analysis methods

#### Palmer Drought Severity Index (PDSI) calculation

The PDSI [28] was computed with the Thornthwaite equation [29] using a self-calibrating PDSI implementation that automatically calibrated the behavior of the index at a given location [30]. The PDSI is a measure of dry and wet spells. In this study, we defined the normal condition as PDSI in the range of [-1, 1], mild drought as PDSI in the range of [-2, -1], moderate drought as PDSI in the range of [-3, -2], and severe drought as PDSI in the range of [-4, -3]. We calculated the PDSI in each study site from 1980 to 2010 to identify the average drought conditions across the SAC.

#### Water use efficiency (WUE) calculation

Soil moisture data at each growth period of spring wheat, naked oat, potato, and maize during 1983–2010 were to calculate farmland water consumption (mm) as following equation:

$$ET_{1-i}=\sum_{i=1}^{n}R_{i}L_{i}\left(l_{i1}-l_{i2}\right)+P_{0}+I+S,\;\;\;\;\;\;\left(i=1,2,\cdots ,n\right)$$ (1)

where *ET*_1-i_ refers to water consumption of the *i*th soil layer; *i* is soil layer number; *n* to total soil layers; *R*_*i*_ to soil dry density in the *i*th layer of soil; *L*_*i*_ to soil thickness in the *i*th layer of soil; *l*_i1_ and *l*_i2_ to water content at the first and last stages of the period in the *i*th layer of soil, calculated as a percentage accounting for dry density, respectively; *P*_0_ to effective precipitation; *I* to irrigation during the period; and *S* to supplemental ground water capacity during the period. When the depth of groundwater is >5 m, *I* and *S* can be ignored because the crops were not irrigated.

WUE used in this study was calculated by the following formula [12, 31].

$$WUE=Y/ET_{a}$$ (2)

where *WUE* means water use efficiency (kg ha^-1^ mm^-1^), *Y* is crop yield (kg ha^-1^) and *ET*_*a*_ stands foractual water consumption during the growth period (mm) (i.e. the sum of water consumption for each stage). In this study, *ET*_*a*_ is the sum of *ET*_1-*i*_ (from 0 to 50 cm) calculated by formula (1). As the depth of ground water is more than 5 m in the SAC, *I* and *S* can be ignored, and water consumption (*ET*_*a*_) is calculated using the sum of soil moisture content and precipitation.

#### Statistical analysis

Firstly, we analyze the trends of climate factors (Tave, Tmax, Tmin, and P) from 1980–2010 across the SAC using observations at 38 weather stations. Secondly, we examine the trend and variation of WUEs in spring wheat, naked oat, potato, and maize fields in Wuchuan and Guyuan stations during 1983–2010. We use Mann-Kendall method to test the significance of trend. Pearson Coefficient of Variation (CV) of WUE was used to indicate inter-annual WUE variability. A box plot was used to intuitively demonstrate the discrete level of WUE data. Thirdly, we use panel regression model (PRM) to unravel the cause-effect relationship for changes in WUE. Correlation coefficient was used to test the relationship of WUE and the impact factors (climate factors, fertilizer). First-order derivative of PRM was used to calculate the threshold of climate factors and fertilizer. Finally, we compare the difference of crop WUE in continuing cropping and rotation cropping from 2008–2010 in Wuchuan station.

PRM that relied on information from multiple impact factors was documented to be better at predicting crop responses to climate change than time-series statistical model at each station [32]. To avoid the confounding effects of highly correlated climate variables, three PRMs with different predictors were estimated to quantify the uncertainties in estimating crop WUE responses to major climate variables. The three PRMs were as follows;

$$WUE_{i,t}=\alpha_{i,0}+\alpha_{1}t+\alpha_{2}T_{i,t}+\varepsilon_{i,t}$$ (3)

$$WUE_{i,t}=\alpha_{i,0}+\alpha_{1}t+\alpha_{2}T_{i,t}+\alpha_{3}P_{i,t}+\varepsilon_{i,t}$$ (4)

$$WUE_{i,t}=\alpha_{i,0}+\alpha_{1}t+\alpha_{3}P_{i,t}+\varepsilon_{i,t}$$ (5)

where *WUE*_*i*,*t*_ is annual crop WUE at station *i* in year *t*. *T*_*i*,*t*_ and *P*_*i*,*t*_ represent growing season average temperature (Tmax or Tmin or Tmean) (depends on which is the impact factor in station *i*) and precipitation at station *i* in year *t*. *α*_i,0_ represents an intercept for each station *i*. *α*_*1*_ represents the linear time trend of observed WUEs mainly due to the long-term no-climatic trends including improvement in cultivars, technology, management, and policy during the study period. The contributions of the adaptations such as the improvements in varieties, technology, management and policy to crop WUE were implicitly described by a non-climatic factor linear trend, i.e., Eqs (3),(4) and (5). *α*_*2*_ represents in two PRMs, i.e., Eqs (3) and (4), represents different estimates of WUEs sensitivity to temperature (Tmax or Tmin or Tmean) (depends on which is the impact factor in station *i*). *α*_*3*_ represents in two RM, i.e., Eqs (4) and (5), represents different estimates of WUEs sensitivity to precipitation during a growth period. *ε*_i,t_ is an error term.

For each station, the sensitivity of WUE change to temperature (Tmax/Tmin/Tmean) and precipitation change during a growth period, i.e. the PRM parameter *α*_*1*_ and *α*_*2*_, respectively, was estimated using multiple regression method based on the data on WUE and climate from 1983 to 2010. The sensitivity was further expressed in percentage of actual mean WUE in each station during the study period as;

$$\alpha_{i}/WUE_{mean}\times 100\%$$ (6)

where *α*_*i*_ is PRM parameter *α*_*2*_ and *α*_*3*_. *WUE*_*mean*_ is the actual WUE in each station during 1983–2010. For each site and each crop, the joint impact of climate change on crop WUE during 1983–2010 (expressed in percentage of actual mean WUE) was computed by summing the impacts of changes in temperature (Tmax/Tmin) and precipitation on crop WUE during the study period. All statistical analyses were conducted using SPSS 13.0. Statistical significance was tested using M-K trend test and the two-tailed t-test. Significance (at *p*<0.05) of any differences for each crop, and different periods were determined using one-way ANOVA.

## Result

### Historical changes in climate factors and fertilizer

**Fig 2:**
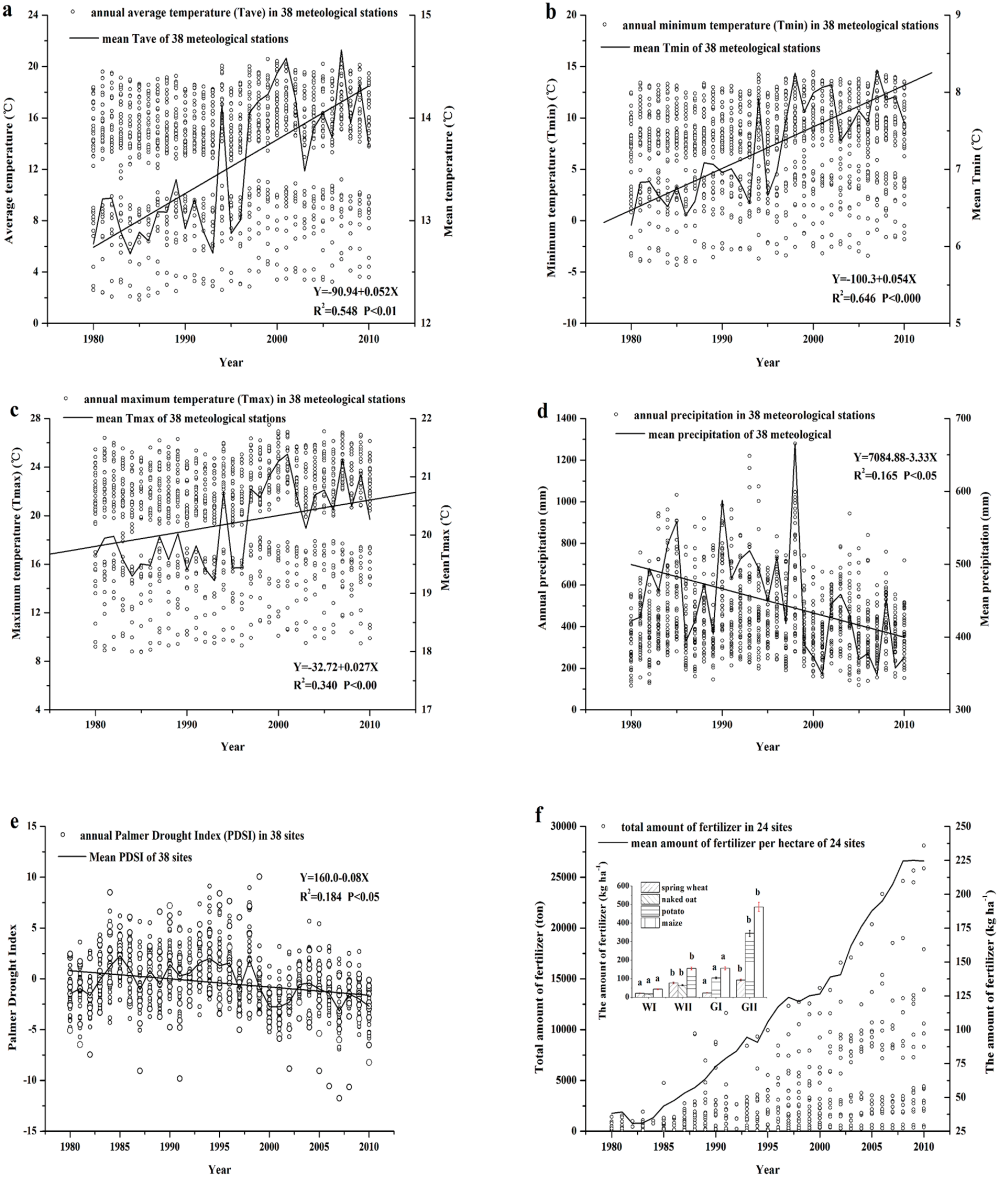
Temporal changes in average temperature (a, °C), minimum temperature (b, °C), maximum temperature (c, °C), precipitation (d, mm), Palmer Drought Severity Index (e), and the fertilization (f, kg ha^-1^) during 1980–2010 in semiarid area of northern China (SAC). The inset in f illustrates differences of fertilizer in spring wheat, naked oat, potato and maize in Wuchuan (W) and Guyuan (G) in stage I and stage II. Columns labeled with the different letter are significantly different (P<0.05). P<0.01 represents the 1% level of significance; P<0.05 represents the 5% level of significance.

During 1980–2010, the SAC experienced a significant warming-drying trend (WDT) during the crop growing season (from May to September) (Fig 2) (see S1 Dataset). Average Tave, Tmin, and Tmax during growth season were 13.5°C, 7.3°C, and 20.2°C, respectively (Table 1). The significant rise in Tave, Tmin and Tmax (Fig 2a, 2b and 2c) per decade was 0.5°C, 0.5°C, and 0.3°C(*P*<0.05), respectively. Compared to those in the period 1983–1999 (stage I), Tave, Tmin, and Tmax all increased by 0.9°C during 2000–2010 (stage II), respectively. During this whole study period 1983–2010, average precipitation was 447.4 mm (Table 1) and significantly decreased in the crop-growing season (Fig 2d). The decrease in precipitation per decade was 33.3 mm (*P*<0.05). Precipitation during growing season decreased by 18.3% in stage II than that in stage I. Fig 2e showed that during 1983–1996, the PDSI were mostly positive that means no drought occurred in this period, while in stage II all PDSI values were negative (around -0.81 to -3.17) which suggests the occurrences of moderate drought in this period. These results indicated that significant WDT occurred during the crop growing season (from May to September) in stage II in the SAC.

**Table 1:** Changes in temperature, precipitation, and fertilizer application crop growth period during 1980–2010.

Factors	Mean	Trend	Highest positive anomaly	Highest negative anomaly
**Tave**	13.5±0.1°C	0.05°Ca^-1^ **	1.2°C	-0.8°C
**Tmax**	20.2±0.1°C	0.03°Ca^-1^ **	1.2°C	-1.0°C
**Tmin**	7.3±0.1°C	0.05°Ca^-1^ **	1.0°C	-1.0°C
**P**	447.4±13.1 mm	-3.33 mm a^-1^ *	218.0 mm	-99.5 mm
**F**	111.7±11.4 kg ha^-1^	6.83 kg ha^-1^ **	113.3kg ha^-1^	-80.9 kg ha^-1^

* Represents the 5% level of significance.

** Represents the 1% level of significance.

Fertilizer application in the SAC experienced a significant rise of 68.3 kg ha^-1^ per decade from 1980 to 2010 (Fig 2f), with an average fertilizer rate of 110.0 kg ha^-1^ among various crops (Table 1) (see S1 Dataset). Average fertilizer input across all crops in the whole region in the stage II was 1.5 times higher than those in the stage I. Average fertilizer usage rate of spring wheat, naked oat, and potato in Wuchuan station increased approximately by 32.3 kg ha^-1^, 26.9 kg ha^-1^ and 64.7 kg ha^-1^ per decade, respectively. Average fertilizer usage rate of spring wheat, potato and maize in Guyuan station increased approximately by 44.4, 157.6, and 219.1 kg ha^-1^ per decade, respectively. Compared to those in the stage I, fertilizer inputs of spring wheat, naked oat, potato and maize increased by 2.1–2.9 times in the stage II. The orders of fertilizer usage to crops are: maize>potato>spring wheat>naked oat.

### The historical change of crop WUE

**Fig 3:**
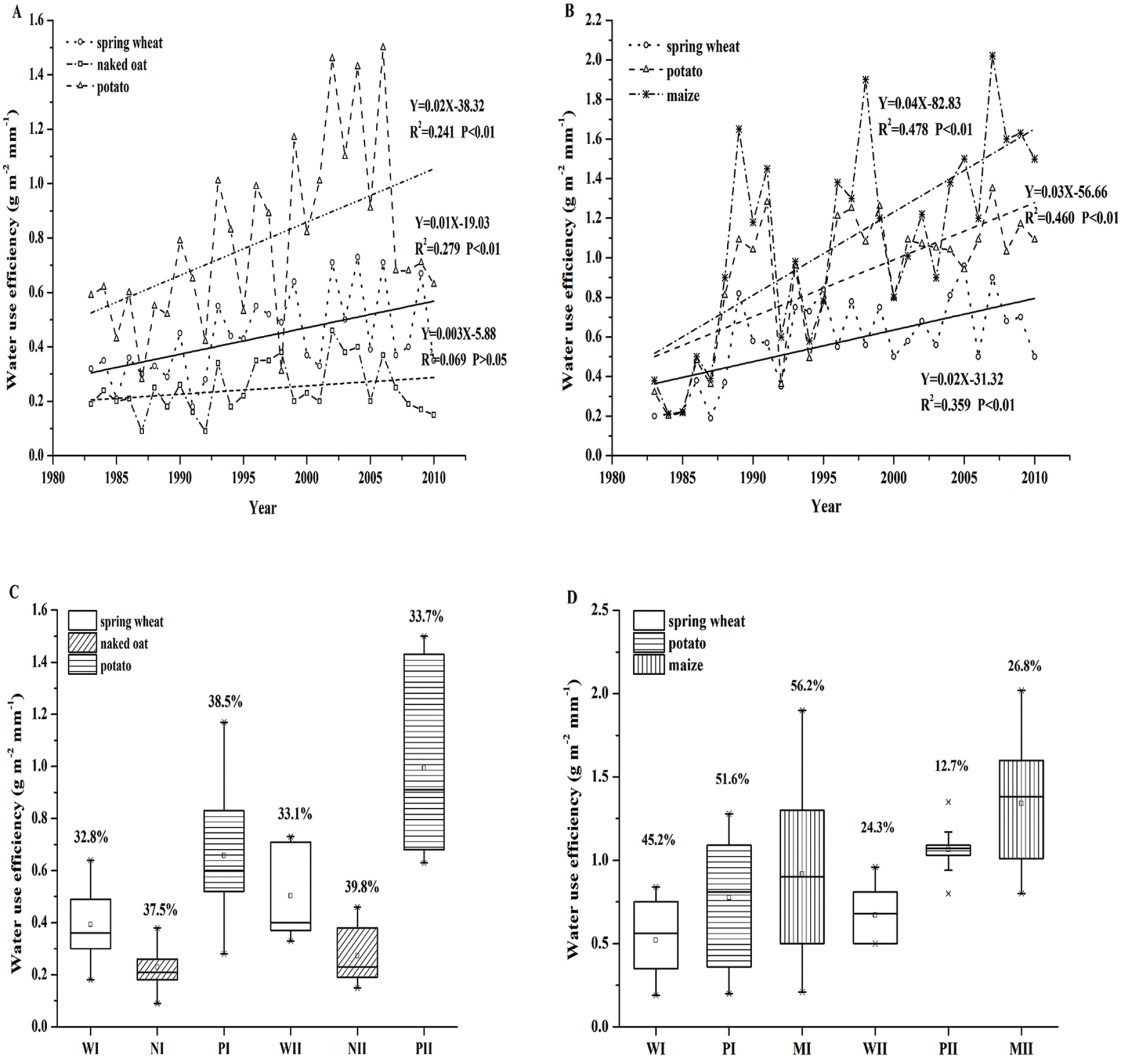
Changes of WUEs of spring wheat (W, black), naked oat (N, red), potato (P, blue), and maize (M, yellow) in Wuchuan and Guyuan from 1983 to 2010, respectively. P<0.01 represents the 1% level of significance; P<0.05 represents the 5% level of significance. A and B refer to the change trend of crop WUE from 1983–2010 at Wuchuan and Guyuan, respectively; C and D refer to the discrete level of WUE at Wuchuan and Guyuan, respectively. In the Fig C and D, “Minimum value”, “1/4 percentile value”, “Median (spot)”, “3/4 percentile value”, “Maximum value” are presented from bottom to top, which is the same case throughout this study. Numbers above box plots are coefficient of variation (CV) of WUE of different crop during the 1980s, the 1990s and the 2000s. I means the period of 1983–1999; II means the period of 2000–2010.

WUEs of spring wheat (*P*<0.01), naked oat (*P*>0.05),and potato (*P*<0.01) in Wuchuan increased significantly by 0.10, 0.03, and 0.20 g m^-2^ mm^-1^ per decade, respectively (Fig 3A) (see S2 Dataset). The WUE increased by 27.7%, 19.2%, and 51.1% in stage II than those in stage I, respectively. WUEs of spring wheat, potato, and maize in Guyuan increased significantly by 0.20, 0.30, and 0.40 g m^-2^ mm^-1^ per decade, respectively (Fig 3B) (see S2 Dataset). The WUE improved of 28.7%, 37.3%, and 46.2% in stage II than those in stage I, respectively. However, compared to CV in stage I, the stability of WUE of spring wheat, naked oat, and potato in stage II in Wuchuan increased by -1.1%, -6.1%, and 12.4%, respectively, with the low-WUE range (0–25% of total WUE interval) significantly (*P*<0.05) from 0.10–0.50 to 0.2–0.70 g m^-2^ mm^-1^ and the high-WUE range (75–100% of total WUE interval) significantly (*P*<0.05) from 0.28–1.30 to 0.40–1.50 g m^-2^ mm^-1^ in stage II (Fig 3C). The stability of spring wheat, potato, and maize in Guyuan increased by 46.3%, 75.4%, and 52.3% in stage II than those in stage I, respectively (Fig 3D). These results showed that WUE increased significantly (*P*<0.05) under the conditions of warm and drought condition with increased fertilization. It indicated that climate change (e.g. warm drought) and high level of fertilizer had significantly improved crop WUE in the SAC over the past 30 years and crop resilience in order to actively tolerate adverse environment stress.

**Fig 4:**
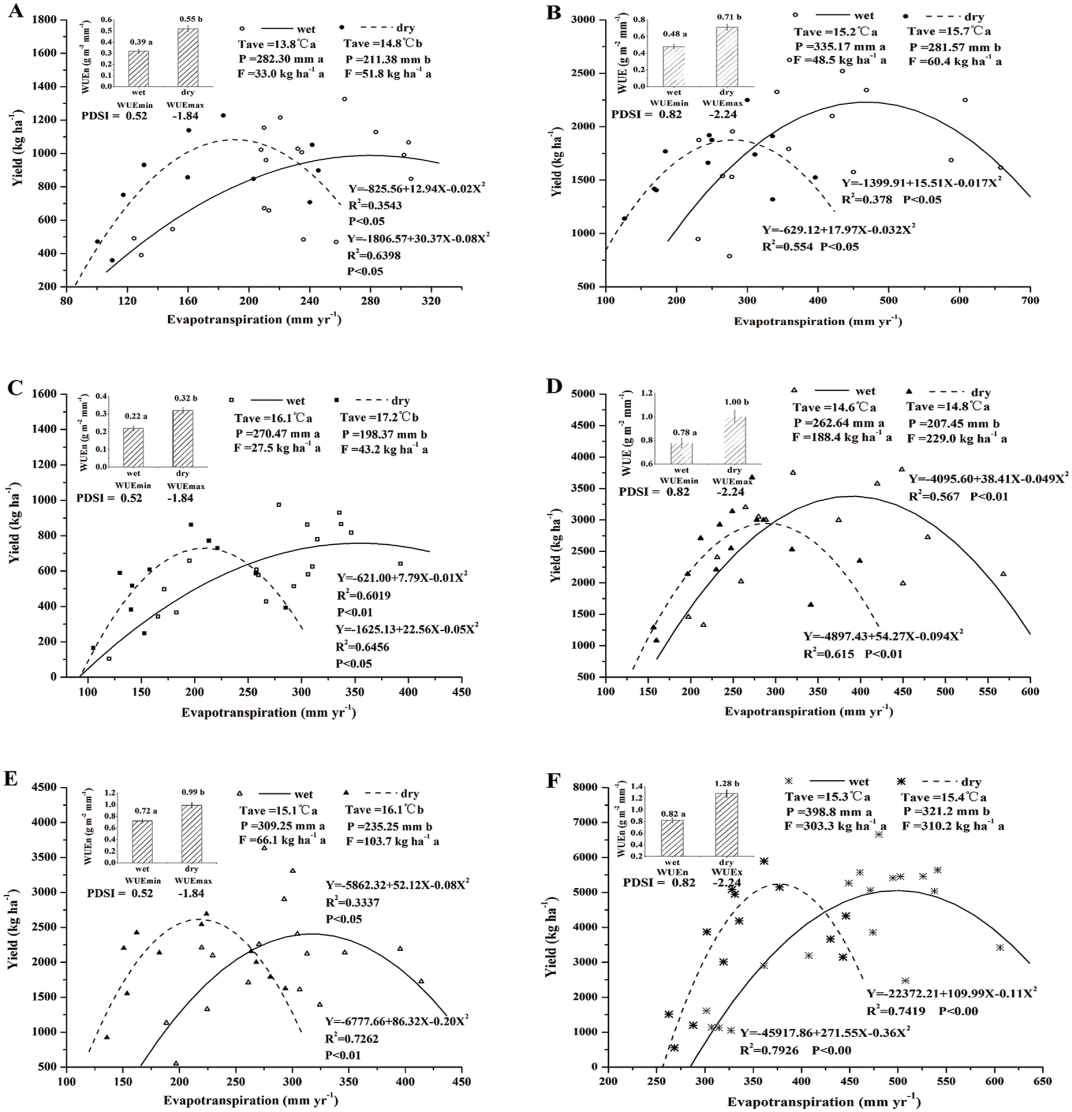
Crop resilience of spring wheat, naked oat, potato, and maize in warm-wet environment (black dot) and warm-dry environment (red dot). A, C and E is spring wheat, naked oat and potato in Wuchuan, respectively. B, D and F is spring wheat, potato and maize in Guyuan, respectively. Minimum (WUEmin) and maximum (WUEmax) (slope of the Yield/ET) in the wetter and drier years, respectively, are based on all crops for multi-year study periods. The insets illustrate the differences in WUEmin and WUEmax with mean Tmax, Tmin, PDSI, and fertilizer for each study periods. Columns labeled with the different letter are significantly different (P<0.05).

As a test of crop resilience, a comparison was made for WUE in the wetter years (PDSI≥-1.00) and in the drier years (PDSI≤-1.00) (Fig 4) (see S3 Dataset). Given the fertilizer inputs were similar in magnitude in the drier and the wetter years, the WDT could be the most crucial impact factor influencing crop WUE. Our results suggest that the lowest WUE (WUEmin) for all crops was usually found in the wetter years. Furthermore, WUE decreased steadily as *ET*_*a*_ increased and kept in a low and stable level when the *ET*_*a*_ reached a threshold and crop yield reached to a maximum value. Beyond this threshold, crop yield would not increase as *ET*_*a*_ changes, implying that redundant moisture would evaporate instead of being utilized by crop. This *ET*_*a*_ threshold in spring wheat, naked oat, and potato field was approximately 280.2, 389.5, and 325.8 mm in Wuchuan, respectively. The *ET*_*a*_ threshold in spring wheat, potato, and maize field was approximately 456.2, 391.9, and 500.0 mm in Guyuan, respectively.

For the drier years, a new curve Yield/*ET*_*a*_ relation was found. Within the certain threshold, higher crop WUE in drier years increased significantly (*P*<0.01) with drought to a maximum WUE (WUEmax) in order to avoid yield declining. The significant (*P*<0.05) increase from WUEmin to WUEmax of spring wheat (Fig 4A), naked oat (Fig 4C), potato (Fig 4E) in Wuchuan is 41.0%, 45.5%, and 37.5%, respectively. The significant (*P*<0.05) increase from WUEmin to WUEmax of spring wheat (Fig 4B), potato (Fig 4D), maize (Fig 4F) in Guyuan is 48.6%, 29.0%, and 55.5%, respectively. The results suggest that the difference of crop WUEmax and WUEmin ranged from 29.0%-55.5% in the SAC. The *ET*_*a*_ thresholds of spring wheat, naked oat and potato maize in Wuchuan in the drier years were 189.8, 200.0, and 225.0 mm, respectively. The *ET*_*a*_ thresholds of spring wheat, potato, and maize in Guyuan in the drier years were 280.8, 288.7, and 377.2 mm, respectively. However, this resilience would collapse as continuing WDT with the same level of fertilizer. These results showed that during the drier years, there was a crop adjustment in WUE that increased with drought intensity, thus sustaining production at near late-twentieth-century levels during prolonged drought; in the wetter years, the sites exhibited an ability to resist the disturbances associated with the early twenty-first century drought and retained the same sensitivity of yield to water availability. It indicated that crop resilience is not only the capacity to tolerate adverse environment stress (e.g. warm drought), but also to actively respond to subsequent periods of favorable circumstance (e.g. water balance) within the threshold.

### Impact of climate change on crop WUE

#### Impact of temperature on crop WUE

Pearson correlation analyses revealed that the WUEs of the three crops in Wuchuan were all significantly positively correlated with crop growing season Tmax and fertilizer, but significantly negatively correlated with growing season precipitation (Table 2). The WUEs of spring wheat, potato, and maize in Guyuan were all significantly positively correlated with Tmin and fertilizer, while also significantly negatively correlated with growing season precipitation (Table 2). These results suggested that Tmax, Tmin, precipitation, and fertilizer were the important factors affecting crop WUE in the SAC.

**Table 2:** Correlation coefficients of water use efficiency of spring wheat, naked oat, potato, and maize with weather factors and fertilizer in the semi-arid area of northern China during 1983–2010 periods.

site	crop	Tave	Tmin	Tmax	P	F
**Wuchuan**	**Spring wheat**	0.208	0.204	0.396*	-0.437*	0.562**
	**Naked oat**	0.119	0.026	0.375*	-0.402*	0.389*
	**Potato**	0.231	0.100	0.509**	-0.452*	0.573**
**Guyuan**	**Spring wheat**	0.376*	0.400*	0.305	-0.400*	0.553**
	**Potato**	0.349	0.427*	0.333	-0.620**	0.603**
	**Maize**	0.608**	0.55**	0.519**	-0.523**	0.662**

Tave means annual average temperature; Tmin means minimum temperature; Tmax means maximum temperature; P means precipitation; F means fertilizer usage per ha.

** represents the 1% level of significance;

* represents the 5% level of significance.

**Fig 5:**
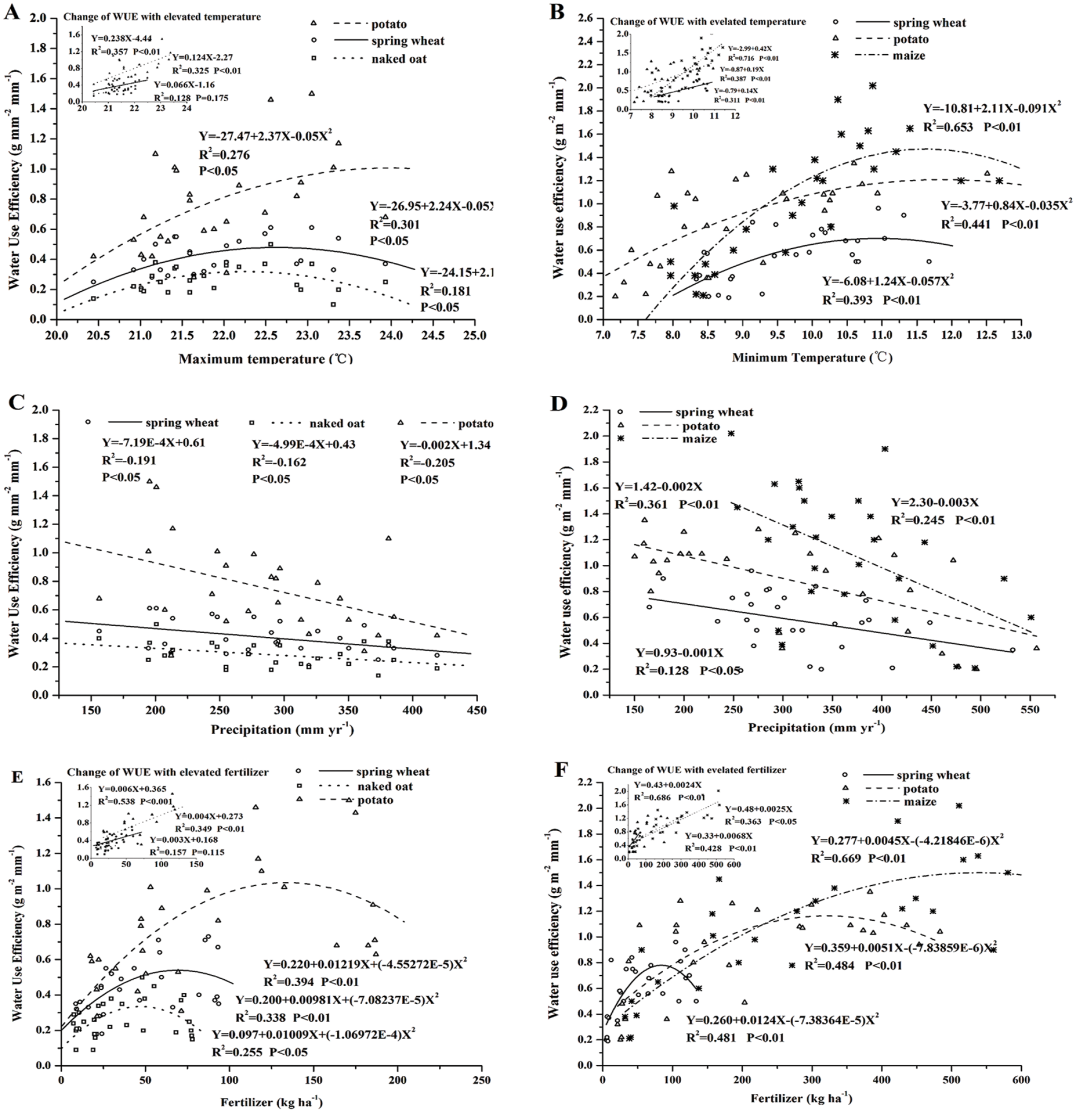
Relationship of crop WUE and Tmax, Tmin, precipitation, and fertilizer during 1983–2010 in the SAC. A, C and E is the relationship of WUE and Tmax, precipitation and fertilizer in Wuchuan, respectively. B, D and Fis the relationship of WUE and Tmin, precipitation, and fertilizer in Guyuan, respectively. The insets illustrate the change of WUE with elevated temperature and fertilizer usage (within the threshold). *P*<0.01 represents the 1% level of significance; *P*<0.05 represents the 5% level of significance.

The relationship curves between Tmax, Tmin, and WUE from 1983 to 2010 for each crop indicated that the WUEs of wheat, naked oat, potato, and maize showed a significant positive trend as Tmax and Tmin rose within the certain threshold (Fig 5A and 5B) (see S4 Dataset). The Tmax threshold of spring wheat, naked oat, and potato in Wuchuan was 22.4, 21.9, and 23.7°C, respectively. The inset in Fig 5A showed that, within Tmax threshold of 22.4°C, wheat WUE increased by 0.12 g m^-2^ mm^-1^ °C^-1^; within 21.9°C, naked oat WUE increased by 0.07 g m^-2^ mm^-1^ °C^-1^; within 23.7°C, potato WUE increased by 0.24 g m^-2^ mm^-1^ °C^-1^. The Tmin threshold of spring wheat, potato, and maize in Guyuan is 10.9, 12.0, and 11.6°C, respectively. The inset in Fig 5B showed that, within Tmin threshold of 10.9°C, wheat WUE increased by 0.14 m^-2^ mm^-1^ °C ^-1^; within Tmin threshold of 12.0°C, potato WUE increased by 0.19 m^-2^ mm^-1^ °C ^-1^; within Tmin threshold of 11.6°C, maize WUE increased by 0.42 m^-2^ mm^-1^ °C ^-1^. However, the single linear WUE/Tmax and WUE/Tmin relation would collapse with continuing warming. This loss of resilience associated with dieback would probably be severer in wheat and naked oat.

#### Impact of precipitation on crop WUE

Crop WUE had a significant negative correlation with precipitation during the growing season over the study period (Fig 5C and 5D) (see S4 Dataset). A decline of 1 mm in precipitation in Wuchuan would increase WUEs by 7.19×10^−4^, 4.99×10^−4^, and 2.0×10^−3^ g m^-2^ mm^-1^ for spring wheat, naked oat, potato, respectively (Fig 5C). A decline of 1 mm in precipitation in Guyuan would increase WUEs of spring wheat, potato, and maize by 0.001, 0.002, and 0.003 g m^-2^ mm^-1^, respectively (Fig 5D). This finding showed that crop WUE would improve as drought stress increase in the SAC over the study period. Xiao et al. [12] and Campos et al. [9] reported that the tolerance of the species to drought stress is robust despite extended perturbation by low precipitation and the increase in water available for vegetation production with increasing precipitation is partially consumed by non-biological components of the hydrological cycle (that is, runoff and deep drainage). In SAC, our results suggested that crops trend to retain their intrinsic sensitivity to water availability during prolonged drought conditions.

#### Impact of the warming-drying trend on crop WUE

**Fig 6:**
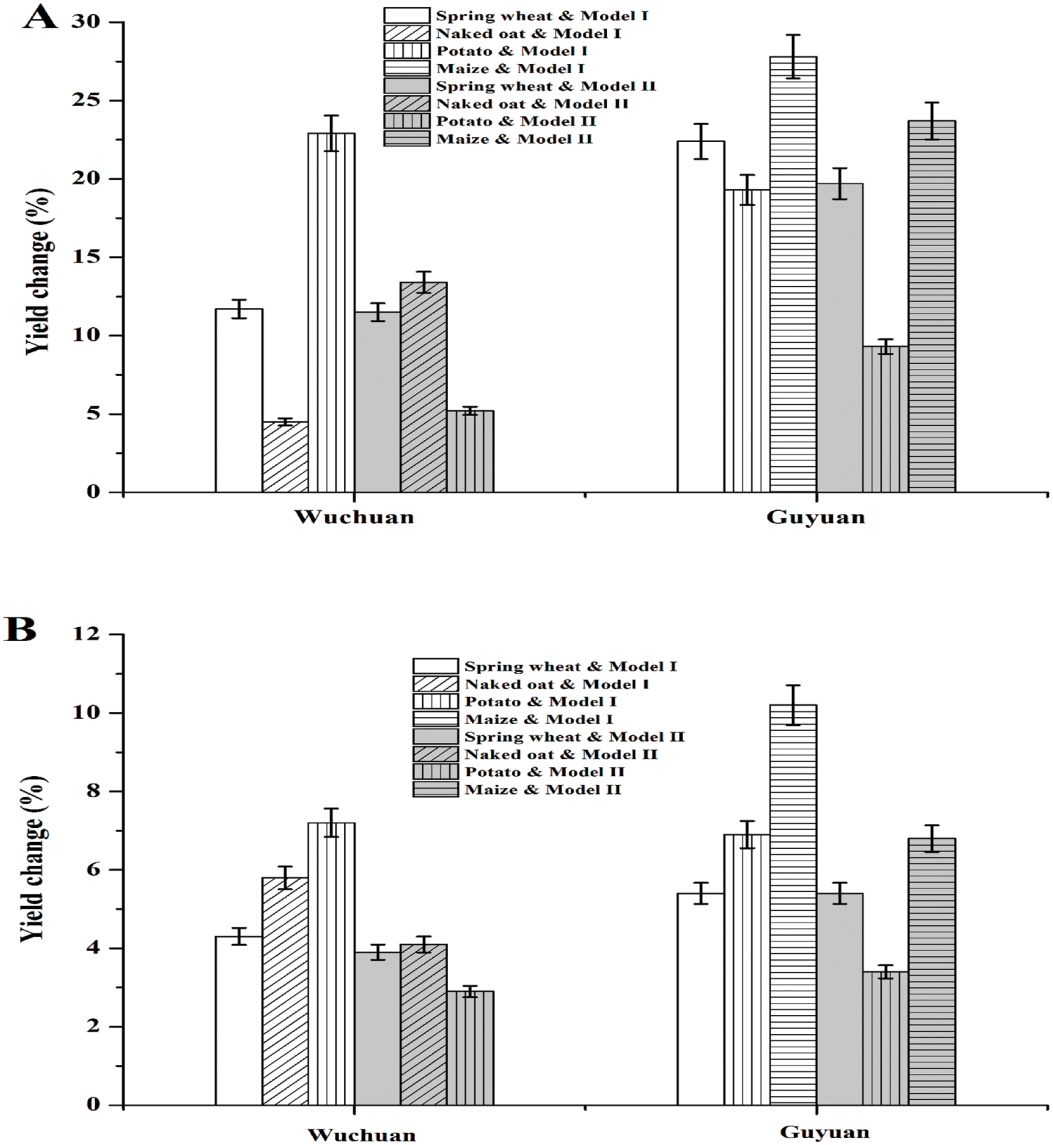
Estimated crop yield changes. Crop yield changes of spring wheat, naked oat, potato, and maize by two panel regression models (PRM) for 1°C increase in temperature (A) and 10% decrease in precipitation (B) during crop growing season in Wuchuan and Guyuan from 1983–2010.

The estimates on sensitivity of crop WUE (spring wheat, naked oat, potato, and maize) to the WDT from the two PRMs were generally consistent (Fig 6) (see S5 Dataset). For each 1°C increase in Tmax during 1983–2010 periods in Wuchuan, WUE of spring wheat, naked oat, and potato increased by 11.6%, 8.9%, and 14.1%, respectively (Fig 6A); as well as for 1°C increase in Tmin during 1983–2010 periods in Guyuan, WUE of spring wheat, potato and maize increased by 21.1%, 14.3%, and 25.7%, respectively (Fig 6A). For each 10% decrease in precipitation during 1983–2010 periods in Wuchuan, WUE of spring wheat, naked oat and potato increased by 4.1%, 4.9%, and 5.0%, respectively (Fig 6B); similarly, 10% decrease in precipitation during 1983–2010 periods in Guyuan, WUE of spring wheat, potato and maize increased by 5.4%, 5.2%, and 8.5%, respectively (Fig 6B). Therefore, observation changes in Tmax and precipitation jointly increased WUE of spring wheat, naked oat, and potato by 15.3%, 17.2%, and 8.1% in Wuchuan during 1983–2010, respectively; changes in Tmin and precipitation jointly increased WUE of spring wheat, potato, and maize by 25.0%, 12.8%, and 30.6% in Guyuan during 1983–2010, respectively (Fig 6).

### Impacts of agronomic practices on crop WUE

#### The influence of fertilizer inputs on crop WUE

The WUEs of spring wheat (*P*<0.01), naked oat (*P*>0.05), potato (*P*<0.05), and maize (*P*<0.01) from 1983 to 2010 showed a significant increasing trend within the fertilizer threshold (Fig 5E and 5F). Through the first derivative (dy/dx), the fertilizer thresholds of spring wheat, naked oat, and potato in Wuchuan were 69.7, 46.0 and 137.2 kg ha^-1^, respectively (Fig 5E). The inset in Fig 5E showed that within fertilizer threshold of 69.7 kg ha^-1^, wheat WUE increased by 0.004 g m^-2^ mm^-1^ kg^-1^ ha^-1^; within 46.0 kg ha^-1^, naked oat WUE increased by 0.003 g m^-2^ mm^-1^ kg^-1^ ha^-1^; within 137.2 kg ha^-1^, potato WUE increased by 0.006g m^-2^ mm^-1^ kg^-1^ ha^-1^. The fertilizer thresholds of spring wheat, potato and maize in Guyuan were 84.0, 325.3, and 533.4 kg ha^-1^, respectively (Fig 5F). The inset in Fig 5F showed that, within 84.0 kg ha^-1^, spring wheat WUE improved of 0.007 g m^-2^ mm^-1^ kg^-1^ ha^-1^; within 325.3 kg ha^-1^, potato WUE improved of 0.003 g m^-2^ mm^-1^ kg^-1^ ha^-1^; within 533.4 kg ha^-1^, maize WUE improved of 0.002 g m^-2^ mm^-1^ kg^-1^ ha^-1^. It showed that, within the fertilizer threshold, 10% increase in fertilizer could enhance WUE of spring wheat, naked oat, and potato in Wuchuan by 6.4%, 5.6%, and 10.4%, respectively; 10% increase in fertilizer could enhance WUE of spring wheat, potato, and maize in Guyuan by10.2%, 11.0%, and 9.8%, respectively. However, the single linear WUE/Fertilizer relation would collapse with continuing increasing fertilization.

Table 3 showed the different effect of fertilizer on WUE in the wetter years and the dry years. The difference of fertilizer between stage I and stage II is significant in both wet and dry years, while the difference of crop WUEs between stage I and stage II was significant in wetter years but non-significant in drier years. It indicated that impacts of fertilizer on WUE in warm-wet environment is stronger than that in warm-dry environment and WUE would not be sensitive to high fertilizer in severe warm-dry environment.

**Table 3:** Impact of the warming-drying trend (WDT) and the amount of fertilizer on water use efficiency in wetter and drier years in different periods in semiarid area of northern China (SAC).

site	year	crop	period	Tmax	Tmin	PDSI	Fertilizer	WUE
				(°C)	(°C)		(kg ha^-1^)	(g m^-2^ mm^-1^)
**Wuchuan**	**wetter-year**	**spring wheat**	I	21.4 a	-	1.29	19.5 a	0.30 a
			II	21.5 a	-	-0.52	80.4 b	0.56 b
		**naked oat**	I	23.5 a	-	1.29	16.3 a	0.14 a
			II	23.6 a	-	-0.52	67.0 b	0.30 b
		**potato**	I	22.6 a	-	1.29	39.0 a	0.64 a
			II	22.7 a	-	-0.52	160.8 b	1.01 b
**Wuchuan**	**drier-year**	**spring wheat**	I	21.8 a	-	-1.36	25.9 a	0.45 a
			II	23.0 b	-	-2.23	73.4 b	0.61 a
		**naked oat**	I	23.8 a	-	-1.36	21.6 a	0.32 a
			II	25.1 b	-	-2.23	61.2 b	0.38 a
		**potato**	I	22.0 a	-	-1.36	51.8 a	0.85 a
			II	24.2 b	-	-2.23	146.9 b	1.11 a
**Guyuan**	**wetter-year**	**spring wheat**	I	-	8.2 a	1.16	25.9 a	0.38 a
			II	-	10.5 b	0.27	83.7 b	0.61 b
		**potato**	I	-	8.8 a	1.16	112.1 a	0.63 a
			II	-	9.2 a	0.27	310.2 b	1.03 b
		**maize**	I	-	8.6 a	1.16	168.2 a	0.53 a
			II	-	10.3 b	0.27	439.1 b	1.24 b
**Guyuan**	**drier-year**	**spring wheat**	I	-	9.4 a	-1.84	37.0 a	0.68 a
			II	-	10.6 b	-2.46	107.0 b	0.78 a
		**potato**	I	-	9.2 a	-1.84	160.5 a	0.95 a
			II	-	9.4 a	-2.46	399.3 b	1.09 a
		**maize**	I	-	9.2 a	-1.84	240.8 a	1.19 a
			II	-	10.3 b	-2.46	566.8 b	1.43 a

I means the years of 1983–1999; II means the years of 2000–2010. Columns labeled with the different letter are significantly different (*P*<0.05).

#### The influence of cropping pattern on crop WUE

The results show large differences of WUE in rotation (millet-potato and rape-potato rotation) and continuous cropping (millet, rape, and potato) (Table 4) (see S6 Dataset). The experiment data from 2008–2010 indicated that, although water consumption of millet and rapeseed in rotation cropping increased by 3.8% and 7.4% than that in continuous cropping, crop yield increased by 10.0% and 104.9%, respectively; while water consumption of potato in rotation cropping was 24.5% less than that in continuous cropping and yield was 10.2% more than that in continuous cropping. Therefore, crop WUEs in rotation cropping were higher than those in continuous cropping. Comparing with WUE in continuous cropping, millet WUE in rotation cropping during 2008–2010 increased by 80.6%, rape WUE increased by 92.9%, potato WUE increased by 19.5%. These results indicated that rotation cropping was in favor of improving crop WUE than continuous cropping.

**Table 4:** The difference of water use efficiency (millet, rape, and potato) in rotation and continuous cropping during 2008–2010.

Crop	Cropping pattern	SM		P	WC	Y	WUE
		SMI(mm)	SMII(mm)	(mm)	(mm)	(kg ha^-1^)	(g m^-2^ mm^-1^)
**Millet**	**R**	157.3±3.6 a	132.5±5.2 a	278.4±11.1	299.8±13.5 a	9577.5±1888.5 a	3.16±0.62 a
	**C**	185.5±22.2 a	175.2±22.4 a	278.4±11.1	288.7±9.4 a	5048.0±1076.8 b	1.75±0.37 b
**Rapeseed**	**R**	138.9±8.6 a	88.4±14.2 a	279.3±12.0	329.8±17.5 a	1769.5±469.4 a	0.54±0.15 a
	**C**	119.2±10.2 a	91.4±14.2 a	279.3±12.0	307.1±21.8 a	863.4±152.3 b	0.28±0.05 b
**Potato**	**R**	118.6±22.4 a	158.7±10.6 a	290.8±9.4	232.5±34.6 a	17923.0±543.0 a	7.43±0.98 a
	**C**	165.2±32.2 a	195.0±17.9 a	290.8±9.4	307.9±26.8 b	16266.2±1919.6 a	6.22±0.47 b

SM means soil moisture;

SMI means soil moisture before sowing;

SMII means soil moisture in harvest period;

P means precipitation;

WC means water consumption;

Y means yield;

WUE means water useefficiency;

R means rotation cropping;

C means continuing cropping.

## Discussion

### Comparison with other studies

The results in this study showed that WUEs of wheat, naked oat, potato, and maize had a significant positive trend as Tmax and Tmin rose within the certain thresholds, yet a significant negative correlation between WUEs and precipitation during the growing season was found over the study period in the SAC. Changes in temperature and precipitation in the past three decades jointly enhanced crop WUE. Elevated fertilizer and rotation cropping could promote this increase. Therefore, it increased by 29.0%-55.5% in warm-dry environment over thirty decades in the SAC compared with crop WUE in warm-wet environment.

The effect of climate change on crop WUE in this study agreed with previous studies. Many studies have been conducted to address the remarkable negative correlation between plant WUE and annual precipitation, and the positive correlation between WUE and temperature within a temperature threshold [7, 8, 9, 10, 11, 12]. However, there still is a study gap that limited our understanding about WUE changes. Firstly, the coupling effect of temperature and precipitation is ignored. Most studies above were mainly focused on single climatic factor (e.g. temperature or precipitation) rather than the effect of multiple climatic factors together on WUE. Secondly, the effect of agronomic practices on crop WUE is ignored. Most studies overstated the effect of climate change on WUE in agriculture system without considering the impact of agronomic managements. Actually, many studies showed that the weather-driven yield was in a declining trend, the management practices (e.g. fertilizer and cultivars) mitigated the weather effects [3, 33]. Moreover, current agricultural activities may affect the physical and biogeochemical interactions within ecosystems [13, 14] and would have the potential to be a main driving force in the change of WUE [17, 18]. This study analyzed the effect of the warming-drying trend, fertilization and cropping pattern on WUE variation, which would fill the gap and might further improve our understanding of the underlying mechanisms of crop WUE response to climate change.

### Impacts of climate change on crop WUE

In this study, 1°C rise in annual maximum/minimum temperature over the study period in the SAC would increase crop WUE within temperature threshold, yet this relation would collapse with continuous warming; 10% decline in annual total precipitation would increase crop WUE by 4.1–8.5%; the effect of the warming-drying trend would enhance crop WUE significantly by 8.1–30.6% in the SAC.

The impact of climate factors on crop WUE has reached a consensus. Rising temperature will affect the plant Eta process by altering the stomatal conductance of a leaf [34, 35]. Increased temperature, under a certain threshold value, improves WUE and will produce a greater increase in net photosynthetic rate [34, 35], compared to the transpiration rate, by increasing the stomatal conductance of the leaves. Temperature over this threshold value will lead to a decrease in water use efficiency due to the rise of evapotranspire [11, 34, 35]. Moreover, as available precipitation in the arid and semiarid areas is the most important factor to control vegetation function, a decline in available precipitation increases the physiological stress and vulnerability of plants. Therefore, the plants have comparatively higher WUE during drought conditions so that they can mitigate the impacts of moisture deficiency and strengthen competitiveness for available moisture [7]. From a plant physiology perspective, plant WUE under drought conditions is mainly affected by stoma and non-stoma limiting factors [36]. The stoma limits WUE by regulating the guard cells in the leaves. When the plant is undergoing mild or moderate moisture stress, the stoma is more sensitive to drought. The reduced stomatal conduction rate, due to the decrease in net photosynthetic rate, will slow down the evaporation rate, thereby improving WUE [36, 37].

However, most researches just focused on the effect of single highly correlated climate variable (e.g. temperature or precipitation) on plant WUE, other correlated climate variables and coupling effect of climate variables (e.g. the joint effect of temperature and precipitation) were ignored. Furthermore, the effects of non-climatic variables such as improvements in varieties, technology management and policy to crop WUE would interfere the results. Ma et al. [1] indicated that increasing temperature would enhance global drought stress, especially in arid and semiarid area. The impact of single climate factor to crop yield might be overstated [38]. The analysis of multiple climate factors to crop yield should be considered to get comprehensive and accurate result. Moreover, the effect of climate change (e.g. the WDT) significantly affects the crop *ET*_*a*_ in the semiarid area [25]. So it’s necessary to analyze the impact of climate change on crop WUE. In this study, three panel regression models with different predictors were used to quantify the uncertainties in estimating crop WUE to major climate variables, avoiding the confounding effects of highly correlated climate variable and the contributions of non-climatic variables implicitly described by a non-climatic factor linear trend, i.e., *α*_1_ in Eqs (7)-(9). Emphatically, the coupling effect of temperature and precipitation (e.g. the WDT) was considered in this study in order to comprehensively analyze the impact of the WDT on crop WUE.

### Impacts of agricultural practices on crop WUE

The declining trend in weather-driven yield was mitigated by agronomic practices (cultivars, fertilizer application, and cropping pattern) [3, 33]. Moreover, they would have the potential to change crop WUE through affecting the physical and biogeochemical interactions within ecosystems [13, 14, 17, 18]. It is a consensus that fertilization could affect crop WUE significantly. For example, WUE of a maize/cowpea intercrop in middle N level to high N level can be increased compared to the control of NPK application [39]; millet WUE is significantly affected by N levels, as N level is increased to a high level, WUE was higher than that at low and middle N level [40]. However, excessive amount of fertilizer exaggerated warm drought stress as the coupling effect of water and fertilizer [41, 42, 43], so that crop WUE declined and crop resilience disappeared. This decreasing resilience through prolonged warm-dry environment suggests that farmland system are closer to a water threshold which, when crossed, will result in soil drought problem.

Some studies also pay attention to the effect of cropping pattern on crop WUE. For example, Wildy et al. [44] indicates that WUE of a mallee eucalypt in short-rotation coppice cultivation over 2.7 years was higher than that in growing naturally at Kalannie Western Australia. WUE of spring wheat, oat, pea, flax, and potato in rotation cropping in the Plateau of north Hebei Province from 1994–1998 varied significantly by 11.4–46.7% than that in traditional cropping [45].

The data of the studies above are just from short-term experiments. The long-term field observations/experiments data in the SAC showed that, fertilization and cropping pattern change were the main factors that affected crop WUE in agronomic measures. This study has demonstrated that WUE of crops would gradually decrease to a certain level in warm-dry environment and then increase as warming-drying trend became more severe in order to avoid the yield decline, while it would keep to a native stable value in favorable water balance condition. This crop resilience would keep the intrinsic system sensitivity to water availability. These results were consistent with that of other studies [7, 8, 11, 12, 36, 37, 46, 47, 48, 49]. However, we further indicated that increased fertilization could promote the resilience within the threshold value and the impact of fertilizer would be more significant in warm-wet environment. Rotation cropping was favor of increasing crop WUE compared with traditional continuous cropping.

**Fig 7:**
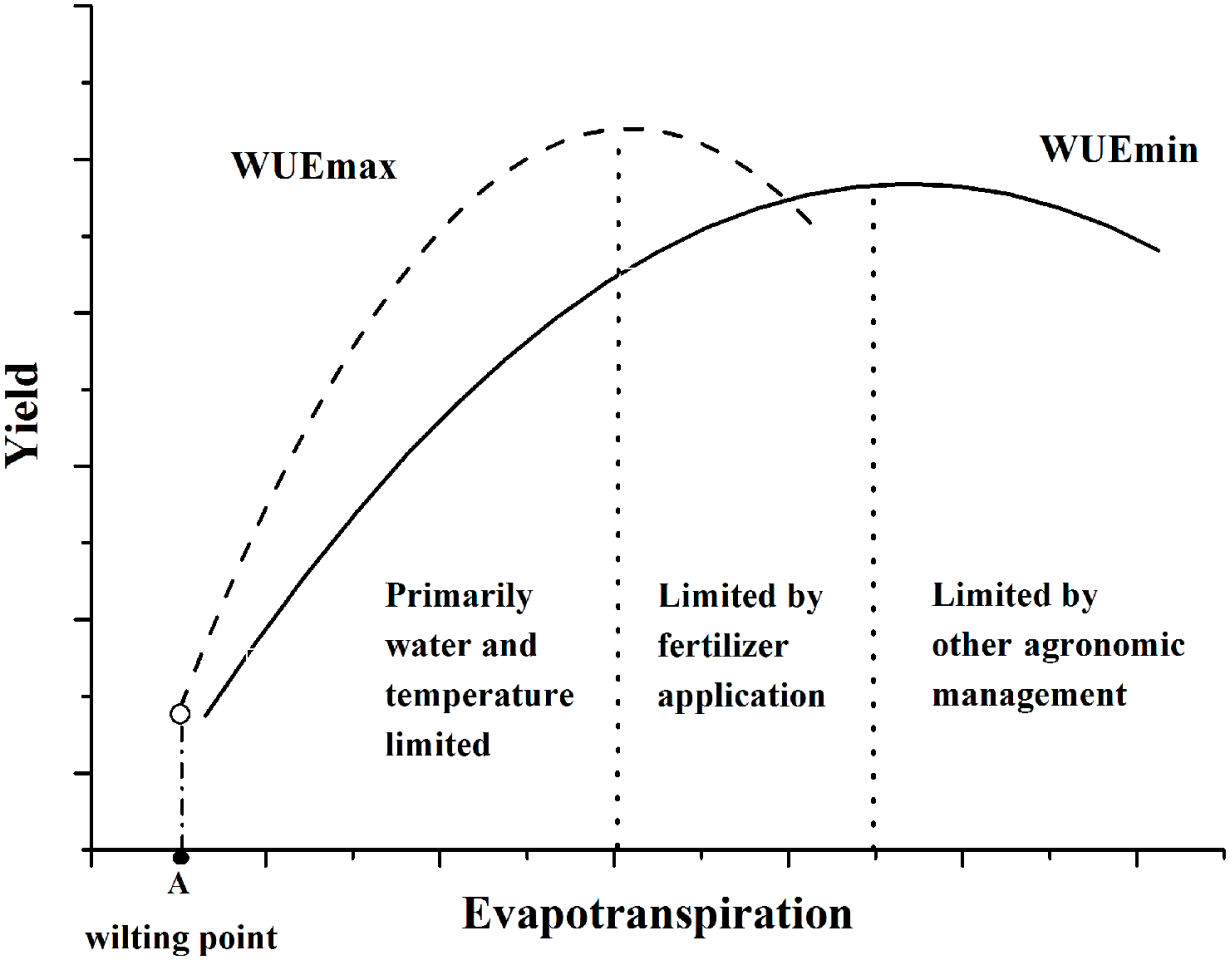
A conceptual model of crop resilience. WUEmin represents the native WUE in the warm-wet environment (black solid line). WUEmax represents WUE affected by warm-dry environment with the same level of fertilization (dash line). WUEmin and WUEmax limited primarily by climate factors and agronomic measures with the arbitrary distinction made here at the most appropriate crop evapotranspiration for illustration only. A refers to crop wilting point of crop.

Our analyses suggest an intrinsic sensitivity of crop to water availability-a capacity to tolerate the drought in warm-dry environment but also to respond to favorable water balance in warm-wet environment. Moreover, moderated fertilization and rotation cropping promoted this resilience. Based on these findings, we constructed a conceptual model of crop resilience (Fig 7). When the crops are limited primarily by water, the WUE will increase to the WUEmax in order to keep the stable level of crop productivity. It indicated that crop capacities and sensitivities of production were maintained by increasing WUE through prolonged warm-dry condition. When precipitation increase gradually and satisfy crop growth, WUE will decrease because fertilization will be the key limited factor to crop production. When fertilizer utilization reaches a highest value (fertilizer threshold), WUE will collapse because production will be limited largely by other agronomic management (e.g. cropping patterns). There is a specific threshold value-wilting point, which indicated the WUE would mutate to be 0 as warm-dry conditions became severe. When the soil moisture content was lower than the wilting point, crop would wither and resilience would collapse.

### Uncertainty and future work

We acknowledge that uncertainties exist in this study due to the inaccuracy of datasets adopted and the lack of other potential factors. For example, the contribution of fertilizer to crop WUE in the different region was not uniform. This study tried to lead an effort to investigate the combination effects of climate condition and agronomic measures on crop WUE. However, there are other potentially important factors such as cultivars that were incomplete or excluded in this study. Previous studies show that the cultivar characteristics that contributed much to yield and WUE increase resistance to biotic/abiotic stresses [4, 50, 51]. The new cultivars with these characteristics were more favorable under climate change conditions than old ones. For example, based on field experiments using leading common wheat cultivars released during 1980–2009 in the North China Plain, Xiao and Tao [52] found that cultivars renewal contributed to yield increase by 2.1–3.6% because of better adaption to warmer climatic conditions [3, 4, 53]. Wei et al. [54] proved that the WUE of wheat varieties with low stomatal conductance in drought-prone rain-fed areas characterized by frequent and long terminal drought increased and high stomata sensitivity to soil drying to make water available during grain filling. Therefore, we made an effort to attribute the relative contributions of cultivars, agronomic management and climate change to crop WUEs change, as described in Eqs (3)–(5). After analyzing the long-term experiment data, however, we found crop varieties did not change much at the stations and half-baked variety records were unavailable for their quantization analysis. In addition, some regions in the SAC are now implementing water conservation methods by using plastic film covering cultivation techniques, water-saving irrigation control, sand cultivation techniques etc. [55], which should be considered as agronomic management in future studies.

## Conclusion

This study investigated the response of crop WUE to climate change and agronomic measures across the semi-arid area of northern China (SAC) during 1983–2010 based on the long-term field observations/experiments data. Our results suggest that the difference of crop WUE in warm-dry environment (WUEmax) and that in warm-wet environment (WUEmin) ranged from 29.0%-55.5%. Change in temperature and precipitation in the past three decades jointly increased crop WUE by 8.1–30.6% in the SAC. We also gained significant results that within the fertilizer threshold, a 10% increase in fertilizer could enhance crop WUE by 5.6–11.0%. Rotation cropping could improve crop WUE with notable increase rates of 19.5–92.9%, relative to traditional continue cropping. These results indicated crop resilience adjusted by WUE is not only able to respond to subsequent periods of favorable water balance but also to tolerate warm drought stress. Reasonable agronomic measures can facilitate this resilience. However, when the warming-drying trend (WDT) became severe, crop resilience would be broken down within the same level of agronomic measures. The study has important implications for the SAC or the similar semi-arid region when trying to mitigate the effects of the WDT. Exploring adaptation strategies in agronomic management is the ultimate purpose for future food security [56]. It is also essential for farmers to consider the availability of resources such as irrigation and fertilizers. However, non-limiting water and fertilizer supply were undesirable in crop production in terms of water shortage and environmental considerations.

## Supporting Information

S1 Dataset Raw data of climate and fertilizer change during 1980–2010. (XLSX)

S2 Dataset Raw data of crop WUE change during 1983–2010. (XLSX)

S3 Dataset Raw data of crop ET and yield in wet and dry years. (XLSX)

S4 Dataset Raw data of climate, fertilizer and WUE in Wuchuan and Guyuan. (XLSX)

S5 Dataset Raw data of panel model. (XLSX)

S6 Dataset Raw data of cropping pattern experiment during 2008–2010. (XLSX)
